# Efficacy of a new nanoemulsion artificial tear in dry eye disease management: Study protocol for a prospective cohort study

**DOI:** 10.1371/journal.pone.0323523

**Published:** 2025-05-09

**Authors:** Xulin Liao, Biyue Guo, Jingfang Bian, Peter H. Li, Jimmy S.H. Tse, William Ngo, Lei Zhou, Thomas Lam

**Affiliations:** 1 Centre for Eye and Vision Research (CEVR), Shatin, Hong Kong SAR, China; 2 Centre for Myopia Research, School of Optometry, The Hong Kong Polytechnic University, Kowloon, Hong Kong **SAR, China**; 3 Research Centre for SHARP Vision (RCSV), The Hong Kong Polytechnic University, Kowloon, Hong Kong **SAR, China**; 4 School of Optometry & Vision Science, University of Waterloo, Waterloo, Ontario, Canada; 5 Department of Applied Biology and Chemical Technology, The Hong Kong Polytechnic University, Kowloon, Hong Kong **SAR, China,**; 6 Research Centre for Chinese Medicine Innovation (RCMI), The Hong Kong Polytechnic University, Kowloon, Hong Kong SAR, China; Jerusalem Multidisciplinary College, ISRAEL

## Abstract

**Background:**

Dry eye disease (DED) is a complex ocular disorder with a significant prevalence worldwide, especially in the Asian population. This study aimed to investigate changes in dry eye symptoms and signs following regular use of a new nanoemulsion eye drop, Systane COMPLETE Multi-Dose Preservative-Free (MDPF), in patients with mild to moderate DED in the Asian population.

**Methods and design:**

This is a prospective cohort study (ClinicalTrials.gov identifier: NCT06188260) that aims to recruit approximately 40 patients from the Asian population suffering from mild to moderate DED. Mild to moderate DED is defined according to the Tear Film and Ocular Surface Society (TFOS) Dry Eye Workshop (DEWS) II diagnostic criteria, including an Ocular Surface Disease Index (OSDI) score between 13–32, and with at least one of the following positive signs: corneal staining, Non-Invasive Tear Breakup Time (NITBUT), or osmolarity. The proposed follow-up period is 3 months. Patients undergo three assessments: baseline before using the eye drops, and follow-up visits after 2 weeks and 3 months regular use of the eye drops (four times daily). The primary outcome is the change in the OSDI score at 2 weeks.

**Discussion:**

The results examine the dry eye symptoms before and after using the new nanoemulsion eye drop, Systane COMPLETE MDPF, in a cohort of mild to moderate DED sufferers. The findings may provide new treatment options for dry eye sufferers with significant clinical implications.

## Introduction

Dry eye disease (DED) is a complex ocular disorder with a prevalence ranging from 5.28% to 33.7% worldwide [1,2]. According to the Tear Film and Ocular Surface Society Dry Eye Workshop (TFOS DEWS) II definition, dry eye is a multifactorial disease of the ocular surface characterized by a loss of homeostasis of the tear film, accompanied by ocular symptoms [3]. The Dry Eye Workshop identified a higher prevalence of DED in the Asian population [4]. Diagnostic methods for dry eye include subjective questionnaires such as the Ocular Surface Disease Index (OSDI) [5], clinical tests including tear break-up time [6], Schirmer’s test [7], corneal and conjunctival staining [8], and assessment of tear film osmolarity [9]. Advanced imaging technologies and biomarkers may also be used for more precise diagnoses [10].

Dry eye is classified into three main types: evaporative dry eye, aqueous-deficient dry eye, and mixed-type dry eye [3]. It is generally believed that most DED patients suffer from both abnormal meibomian gland function and inadequate tear production, rather than from just one distinct cause [11]. If only one of these two broad categories of DED is addressed therapeutically, patients may continue to suffer symptoms and report dissatisfaction with the prescribed treatment. Treatment options for dry eye vary depending on its severity and underlying causes. These include lifestyle modifications, eyelid hygiene, warm compresses, artificial tear supplements, anti-inflammatory therapies, punctal plugs, and advanced treatments including intense pulsed light therapy [11,12]. Various types of artificial tears are available on the market, including preservative-free drops, gel-based formulations, lipid-based drops for evaporative dry eye, and enhanced solutions containing hyaluronic acid to support ocular surface health [11,13]. These products aim to supplement and stabilize the tear film, relieve symptoms, and protect the ocular surface.

Recently, a combined lipid-aqueous nanoemulsion artificial tear solution, Systane COMPLETE Multi-Dose Preservative-Free (MDPF) (Alcon, Fort Worth, TX, USA), has become available. This sterile white emulsion contains propylene glycol, hydroxypropyl guar, mineral oil, dimyristoyl phosphatidylglycerol, polyoxyl 40 stearate, sorbitan tristearate, boric acid, sorbitol, and purified water. Utilizing nano-droplet technology, this artificial tear solution ensures a uniform distribution of lipids, resulting in less blur upon installation [14,15]. This new eye drop is similar in composition to another product from the same company, Systane COMPLETE, which is also propylene glycol-hydroxypropyl guar (PG-HPG) nanoemulsion lubricant eye drops, but the new solution has the advantage of not containing preservatives [16].

A previous study reported that one of the main ingredients in these eye drops, hydroxypropyl guar, enhanced corneal epithelium cell hydration and recovery better in a corneal epithelium model compared to traditional microemulsion eye drops [17]. Another study reported that Systane COMPLETE, which had a similar composition to MDPF, was effective and well tolerated in participants with DED and all its subtypes [18]. However, these results were based solely on patients’ subjective responses without key objective ocular measurements or control groups of normal subjects. A recent review paper indicated that most reports compared the performance of PG-HPG nanoemulsion lubricant eye drops to other formulations, with limited objective outcome measures [16]. Its temporal effects in managing dry eye conditions, especially in the Asian population, were lacking.

This research is the first prospective cohort study of Systane COMPLETE MDPF in an Asian population. In this study, the subjective symptoms and objective ocular surface changes following the recommended dosage of the new nanoemulsion eye drop in DED patients in Hong Kong are investigated. To address gaps in the use of widely accepted objective measurements in published studies, this cohort study applies the up-to-date protocols recommended by the Tear Film and Ocular Surface Society (TFOS) Dry Eye Workshop (DEWS) II [4,9].

One objective of this study is to compare the subjective changes in symptoms using the Ocular Surface Disease Index (OSDI) questionnaire following the recommended use and dosage of Systane COMPLETE MDPF in mild to moderate DED patients. The OSDI questionnaire is administered after 2 weeks of treatment as the primary endpoint. The second objective is to investigate the objective ocular surface changes using modern clinical instruments during the study period.

## Methods

### Study design and ethical approval

The study protocol was developed in accordance with the SPIRIT guidelines (S1 File). This is a prospective cohort study, which was reviewed and approved by the Hong Kong Polytechnic University Institutional Review Board (IRB) (Approval Number: HSEARS20230209004) and adhered to the tenets of the Declaration of Helsinki (S3 File). Research team members and registered optometrists were required to hold a valid certificate for Good Clinical Practice (GCP). Participant recruitment is conducted through open recruitment. The recruitment period started on November 23, 2023, and ended on September 1, 2024. All patients signed the written informed consent (S2 File). Online self-registration and screening were conducted, followed by clinical evaluations at the Optometry clinic, the Hong Kong Polytechnic University (https://www.polyu.edu.hk/so/optometry-clinic/) and/or Centre for Eye and Vision Research (CEVR) at Hong Kong Science Park (https://cevr.hk/).

### Inclusion and exclusion criteria

The inclusion criteria for the study require participants to be aged 20–50 years, have a visual acuity of ≥ 6/9, and exhibit a mild to moderate OSDI score ranging from 13 to 32. Participants are also required to have a positive result in at least one of the following objective tests: ocular surface (corneal and conjunctival) desiccation according to TFOS criteria, a first Non-Invasive Tear Break-Up Time (first-NITBUT) of < 10 seconds, or an osmolarity of ≥ 308 mOsm/L or a difference of > 8 mOsm/L.

Exclusion criteria include active ocular infections, eyelid inflammations or anomalies, uncontrolled or newly diagnosed systemic diseases, or changes in long-term medications within the past six months that could affect the tear profile. Additionally, pregnant or breastfeeding individuals, contact lens wearers who had not discontinued use for at least one month prior to evaluation [19,20], and individuals using artificial tears, eye drops, or systemic drugs known to cause dry eye (e.g., antidepressants, antipsychotics, or systemic corticosteroids) were excluded.

### Sample size calculation

The sample size calculation is based on the OSDI data published by Craig et al. 2021 [21], which detected a 5-point difference in OSDI scores between baseline and endpoint assessments at 1 month, with an expected standard deviation of 6.7 points. The estimate for type 1 error (α) is set at 0.05, and the study aims for statistical power of 0.95 in a paired study design (2-sided). The sample size is adjusted for a 20% discontinuation rate. Approximately 40 subjects are expected to be recruited, with a total of 27 subjects being eligible for completing the study and subsequent data analysis.

### Procedures and interventions

Participant recruitment is conducted through open recruitment on the university social media platform (Facebook), newspaper advertisement or any other open recruitment method. Before enrollment, the details of this study are fully explained, and written consent obtained. Prior to the study, all forms of eye drops and contact lenses must be discontinued and washed out for at least one month. All participants must undergo screening evaluations using OSDI scores. Subjects with mild to moderate DED (OSDI score between 13–32) are included, while those with severe DED excluded from the study.

**Fig 1:**
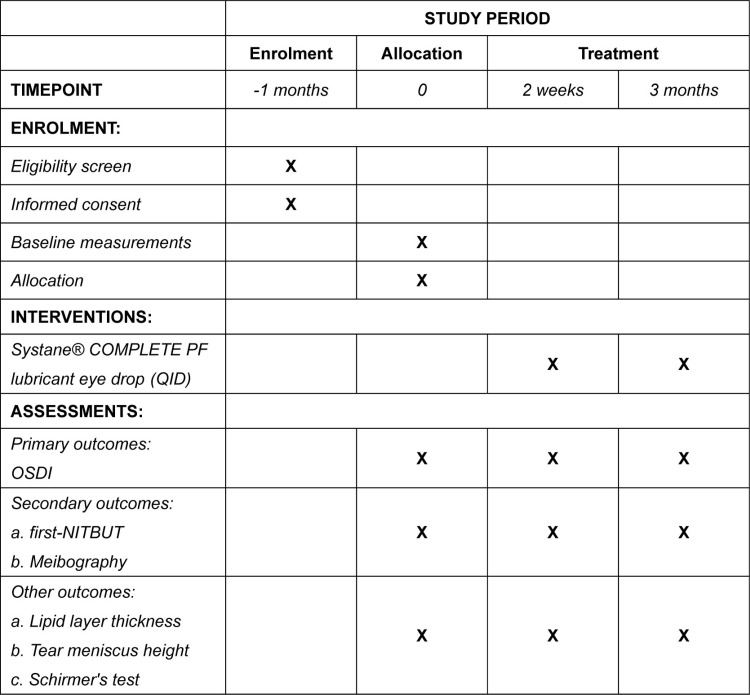
The schedule of enrolment, interventions, and assessments. **Aberrations**: PF, preservative-free; OSDI, Ocular Surface Disease Index; NITBUT, Non-Invasive Tear Break Up Time; QID, four times a day.

After baseline assessments, DED patients receive Systane® COMPLETE MDPF lubricant eye drop to be used according to the manufacturer’s guidelines (four times daily for 3 months, between 9 AM and 9 PM). Objective measurements are scheduled between 4 PM and 7 PM to control for diurnal variations for all follow-up visits. (Fig 1)

### Outcomes and measurements

The primary outcome of the study is the change in OSDI score after a 2-week visit. Secondary outcomes include first-NITBUT and meibography assessments conducted at the 2-week and 3-month visits. Additionally, other exploratory outcomes evaluated include changes in average Lipid Layer Thickness (LLT) from baseline, tear meniscus height (TMH), and the results of Schirmer’s test without anesthesia.

The screening measurements include visual acuity (habitual or best-corrected better than 6/9), assessed using a Snellen chart; OSDI scores (13–32); and any one of the positive results, such as tear osmolarity, NITBUT, or ocular surface staining. At subsequent visits, measurements include NITBUT, meibography, lipid layer thickness (LLT), tear meniscus height, Schirmer’s test, ocular surface staining, and lid margin assessment. All clinical evaluations and equipment are performed in a designated dry eye examination room maintained at a temperature of 23–25°C and approximately 50% humidity. Measurements are conducted on both eyes and taken after participants stayed in the examination room for at least 10 minutes. The examination flow and instruments used in each assessment are listed in Table 1.

**Table 1:** Order of assessment and equipment.

Order of assessments	Examination items	Equipment
1	Dry eye questionnaire	OSDI
2	Tear film lipid layer thickness	IDRA Ocular Surface Analyser (SBM SISTEMI, Italy)
3	Meniscometry	Keratograph 5M (Oculus Optikgerate, Germany)
4	NITBUT	Keratograph 5M (Oculus Optikgerate, Germany)
5	Tear osmolarity	TearLab (TearLab, Escondido, CA, USA)
6	Tear fluid collection	Schirmer strip (I-Dew Tear Strips, Entod Research Cell, UK)
7	Slit-lamp biomicroscopy	Slit-lamp biomicroscopy (DC-4, Topcon, Tokyo, Japan)
8	Infrared Meibography	Keratograph 5M (Oculus Optikgerate, Germany)
9	Non-contact tonometer	Tonometer (TX-10, Canon, Japan)
10	Visual acuity	Snellen chart

**Abbreviations:** OSDI, Ocular Surface Disease Index; NITBUT, Non-Invasive Tear Break Up Time.

#### OSDI questionnaire.

The OSDI questionnaire [5] is administered using the Qualtrics XM platform. The OSDI questionnaire contains 12 questions divided into 3 subscales: ocular symptoms, vision-related function, and environmental triggers. Participants are required to rate each symptom on a 5-point scale according to its frequency. The final score, which ranges from 0 to 100, is calculated, with scores between 13 and 32 classified as mild to moderate DED.

#### Lipid layer thickness (LLT).

The LLT is video-documented for 15 seconds using the IDRA Ocular Surface Analyzer (SBM SISTEMI, Italy) [22]. Three video captures are taken, and the LLT measurements averaged to provide the final LLT value. LLT is categorized as thin (<60 nm), indicating tear instability; normal (60–99 nm), signifying a healthy tear film; or thick (≥100 nm), which may suggest excessive lipid production or compensatory changes [23].

#### Meniscometry.

The tear meniscus height (TMH) measurement is determinedusing the Keratograph 5M (Oculus Optikgerate Gmbh, Wetzlar Germany) [24]. The lower TMH is measured by averaging three measurements taken near the pupil center at the lower meniscus. Participants are instructed to look straight ahead during the measurement, which is captured using infrared LED lighting. The software’s caliper tool is used to measure the TMH for each image captured. A TMH of < 0.2 mm may indicate lacrimal gland deficiency, suggesting reduced tear production and potential aqueous-deficient dry eye [25].

#### Non-invasive tear break-up time (NITBUT).

Tear film stability and regularity are assessed using Placido disk projection with the Oculus Keratograph 5M [6]. The software automatically detects the first and average breakup times, and readings from three independent captures are averaged. All measurements are taken using infrared LED lighting. NITBUT of less than 10 seconds indicates tear film instability [9].

#### Tear film osmolarity.

The clinical osmometer TearLab (Escondido, CA, USA) is used to measure tear film osmolarity [26]. The osmolarity measurement procedure follows manufacturer guidelines. Briefly, calibration is performed daily before use. A tear sample of 50 nL is collected by positioning the osmolarity pen tip at the lower lateral canthus tear meniscus. To prevent stimulating reflex tear secretion, participants are instructed to look in the superior nasal direction during measurement. Readings from each eye and the inter-ocular difference are recorded and analyzed. An abnormal tear film osmolarity value is typically ≥308 mOsm/L or a difference of more than 8 mOsm/L between the eyes, which indicates tear film instability and is commonly associated with dry eye disease [9].

#### Tear fluid collection and analysis.

The Schirmer strip (I-Dew Tear Strips, Entod Research Cell, UK) is used to collect tear samples [7]. Participants are asked to look in the superior nasal direction, and the strip positioned at the lateral canthus. Once placed, participants are instructed to keep their eyes closed during tear sample collection. Approximately 10–20 µ L of tears are collected. The sampled Schirmer strip is heated using an optical frame heater to dry it out and prevent protein degradation. The dried strip is then stored in a 1.5 mL Eppendorf tube. Protein assays are carried out subsequently in the laboratory [27].

#### Slit-lamp biomicroscopy.

Lid margin, lashes, corneal and conjunctival integrity are assessed using slit lamp biomicroscopy (DC-4, Topcon, Tokyo, Japan). Sodium fluorescein and lissamine green dyes are applied to evaluate corneal and conjunctival desiccation,respectively [8]. Staining was recorded and graded using the NEI scale [28] and lid wiper epitheliopathy is evaluated using Korb’s grading [29]. Lid and lash abnormalities are also graded based on a four-point scale [30]. All anterior assessments are photo-documented.

#### Infrared meibography.

The meibomian glands are imaged using near-infrared light with the Oculus Keratograph 5M [31]. Both upper and lower tarsal conjunctiva are assessed and the evaluation of the meibomian gland graded using Meiboscore [32]. The grading scale ranges from 0 to 3 for each eyelid: 0 indicates no loss of meibomian glands, 1 represents less than one-third of the glands lost, 2 indicates one-third to two-thirds of the glands lost, and 3 denotes more than two-thirds of the glands lost. The scores for the upper and lower eyelids are combined, resulting in a total score ranging from 0 to 6 for each eye.

### Safety

This study establishs a set of guidelines for reporting adverse events during the research period. Any incidents involving abnormal clinical findings, symptoms, or illnesses related to the use of eye drops by participants, such as discomfort or allergic responses, whether or not they were considered to be related to the eye drops, are reported and recorded,. The information collected includes a description of the event, the onset time, the clinician’s assessment of severity, the relationship to the intervention, and the time of event resolution. The research management committee evaluates whether participants met the inclusion criteria and whether they should withdraw from the study. Additional visits are arranged to further assess the condition. For dropout patients, the reasons for their dropout are also recorded and assessed and followed up with the participants.

### Statistical analysis

Statistical analysis will be conducted using IBM SPSS (Amonk, New York, USA). Inter-group comparisons of normally distributed continuous measures will be conducted using one-way ANOVA tests, followed by Bonferroni corrections. Non-normally distributed continuous measures will be compared using Kruskal-Wallis tests. Ordinal data and categorical data will be compared using Mann-Whitney tests and Fisher’s exact tests, respectively. Univariate and multivariate logistic regression will be conducted to evaluate the relationship between DED and lifestyle factors. A p-value of less than 0.05 will be considered significant.

## Discussion

This study represents the first prospective cohort investigation of Systane COMPLETE MDPF in Asian patients with mild to moderate DED. Measurements are conducted at three intervals: baseline, 2 weeks, and 3 months, with patients using the product four times daily. The study gathers extensive objective and subjective examination data, providing a dataset for evaluating the efficacy and safety of Systane COMPLETE MDPF in the Asian patient population. There are numerous treatment options for dry eye, including warm compresses [33], traditional Chinese medicine fumigation [34], moisture chamber glasses [35], Intense Pulsed Light [36], and LipiFlow [37]. For severe cases of different types of dry eye, medications such as Cyclosporine [38] are used. However, artificial tears are the most common and widely used eye drops for effectively alleviating dry eye symptoms [39]. Therefore, research on artificial tears is highly necessary and valuable.

Several studies have demonstrated the effectiveness of Systane Complete, an artificial tear, in alleviating dry eye symptoms. Silverstein et al. 2020 showed that Systane Complete provided instant and sustained symptom relief for up to 8 h after a single application and was well tolerated in patients with dry eye disease [18]. Additionally, Pucker et al. 2021 found that Systane Complete was safe for use in contact lens wearers and significantly improved ocular symptoms compared to no treatment after two weeks of use [40]. Hydroxypropyl guar, a key ingredient in Systane COMPLETE MDPF, showed substantial benefits for dry eye in both cellular studies [17] and multiple clinical trials [16,41,42]. POLYQUAD (polidronium chloride) 0.001% is the preservative used in Systane Complete. However, this study focuses specifically on the preservative-free version, Systane COMPLETE MDPF, to evaluate whether it could provide both objective and subjective relief for dry eye patients.

Since Systane COMPLETE MDPF is a relatively new medication, published clinical studies on its use are limited. A recent study performed in USA indicated that Systane COMPLETE MDPF significantly improved the quality of life and reduced dry eye symptoms for digital device users. In contrast, this research primarily involved subjective assessments and a Caucasian population [14]. The current study addresses these gaps by incorporating a wide range of objective evaluations and focusing on an Asian population, thereby providing a more comprehensive understanding of the medication’s efficacy.

This study design does have certain limitations, including exclusion of patients with severe DED and the lack of a randomized controlled trial. Additionally, it is relatively small in scale, and conducted at a single center. Further clinical trials are needed to address these shortcomings. Nonetheless, the current study can contributed valuable insights into the use of Systane COMPLETE MDPF, particularly for a broader patient population, and may help expand treatment options for dry eye patients.

## Conclusion

This study may significantly enhance the understanding of the effects of Systane COMPLETE MDPF in Asian patients with mild to moderate DED. The study may directly benefited DED patients by addressing gaps in current knowledge and provide additional data and options regarding artificial tears for those suffering from this condition. This research may contribute valuable insights and practical solutions to improve the management and treatment of DED.

## Supporting information

S1 File Checklist. SPIRIT 2013 checklist: Recommended items to address in a clinical trial protocol and related documents.
(DOC)

S2 File Information sheet and Consent form. (DOCX)

S3 File Ethics approval. (PDF)

S4 File Ethics committee approved protocol. (DOCX)
