# Supplementary material for: Efficacy of a new nanoemulsion artificial tear in dry eye disease management: Study protocol for a prospective cohort study
Source: PLoS One. 2025 May 9;20(5):e0323523. doi: 10.1371/journal.pone.0323523 (PMC12063801; doi:10.1371/journal.pone.0323523)
Supplement: S2 File — (DOCX) [file pone.0323523.s002.docx]

Research Study Information Sheet

**Title of Project:**

Efficacy of a new nanoemulsion artificial tear in dry eye disease management

**Project Leader:**

Dr Thomas Lam SO

**Project members:**

Dr Jennifer Bian SO

Dr Ella Guo SO

Mr Jimmy Tse SO

Mr Peter Li SO

**Why is the study being performed?**

The aim of this study is to investigate the changes in clinical signs and symptoms of patient with mild to moderate dry eye disease (DED), before and after using this new nanoemulsion artificial tear

**Who can participate in this research study?**

Local Chinese residents aged 20-50 years old; with best-corrected visual acuity ≥ 6/9, mild to moderate Ocular Surface Disease Index score 13-32; any one of the objective test positive (non-invasive tear breakup time/corneal staining/tear osmolarity); tear lipid layer thickness <65nm on both eyes.

**Who can’t participate in this research study?**

With any active ocular infections; Inflammations or anomalies in the eyelid; Uncontrolled, newly diagnosed systemic diseases or with modified long-term medications within 6 months that are known to affect tear profile; Pregnancy and breastfeeding; Contact lens wearers are required to stop contact lenses wear for at least 1 week before the evaluation; Using artificial tears or other eyedrops will be excluded; Taking systemic drugs that may cause dry eye, e.g., Antidepressants/antipsychotics, Systemic corticosteroids.

**What do I have to do?**

If you agree to participate in this study, you will be asked to sign a consent form indicating that you understand the information presented on this sheet. You will be required to provide personal information, including name, date of birth and contact number. If you are eligible for the study, you will be instructed to use this new nanoemulsion artificial tear for 3 months, and given comprehensive dry eye assessment before the treatment, and after 2 weeks and 3 months of treatment. The assessment includes questionnaire filling, tear fluid examination and collection, and eyelid examination. Tear collection will be performed using schirmer strip and eyelid examination required eversion of upper eyelid, which may cause slight discomfort.The estimated assessment time will be 1-1.5hr for each visit.

Is there any benefit or risk in the study?

Benefits: You will be provided with artificial tears for 3 months. You will be given comprehensive dry eye assessment during this period that evaluates the condition and severity of dry eye before and after the treatment. The optometrist could provide you advice on managing the dry eye condition.

Risk: There is no significant risk associated with the assessment procedures of dry eye or usage of artificial tears.

In case of a serious adverse event, please **report to the Principal Investigator/Chief Investigator immediately and the Principal Investigator/Chief Investigator will be required to report it to the PolyU IRB within 48 hours upon the receipt of your report. The insurance will be covered by University Master Insurance plan.**

**Can I withdraw from the study?**

Yes, you can stop participating in the study at any time with no penalty or any prejudice.

**Can I get more information on the study?**

Yes, contact *Dr Ella* Guo and *Mr. Peter Li* and they will try to answer any questions you may have.

**Confidentiality**

Information collected will be kept confidential. Results from the current study may be published in a professional journal but no personal data will be disclosed. Data retrieved will only be accessible by the study staff and subjects will be given coded identification number. Data collected will be analyzed, presented (e.g. at conferences), and published but no personal information will be revealed. Personal information will only be accessed when demographic analysis needed or contacting subject for further information and follow-ups, by authorized staff after PI’s approval. Any relocation of records to off campus storage space will require prior approval from the study investigators. All records will retain in PolyU for five years after completion/termination of the study.

This study has been approved by the Departmental Research Committee (DRC) of the School of Optometry of The Hong Kong Polytechnic University. If you have any complaints about the conduct of this research study, please do not hesitate to contact the the Secretary of the Institutional Review Board (IRB) of The Hong Kong Polytechnic University in writing (institutional.review.board@polyu.edu.hk), stating clearly the responsible person and department of this study as well as the HSESC Reference Number HSEARS20230209004.

**Title of Study**

Efficacy of a new nanoemulsion artificial tear in dry eye disease management

**Informed Consent Form**

| **Have you read the information sheet provided?** | **Yes / No** |
| --- | --- |
| **Have you had an opportunity to ask questions and discuss this study?** | **Yes / No** |
| **Have your received satisfactory answers to all of your questions?** | **Yes / No** |
| **Have you received enough information about the study?** | **Yes / No** |
| **Do you understand that participation is entirely voluntary?** | **Yes / No** |
| **Do you understand that you are free to withdraw from the study** |  |
| **at anytime without having to give a reason****without affecting your future care** | **Yes / No**  **Yes / No** |
| \| **………………………………………………………………**  **Signature of participant** \| **……………………….……………………**  **Signature of Research Staff** \| \| --- \| --- \| \| **………………………………………………………………**  **Name of * participant** \| **……………………….……………………**  **Name of Research Staff** \| \| **………………………………………………………………**  **Date** \| **……………………….……………………**  **Date** \| | |
|  |  |

** Delete as appropriate*

**Reference no: _________________**

**(Office use only)**
