# Supplementary material for: Efficacy of a new nanoemulsion artificial tear in dry eye disease management: Study protocol for a prospective cohort study
Source: PLoS One. 2025 May 9;20(5):e0323523. doi: 10.1371/journal.pone.0323523 (PMC12063801; doi:10.1371/journal.pone.0323523)
Supplement: S3 File — (PDF) [file pone.0323523.s003.pdf]

THE HONG KONG POLYTECHNIC UNIVERSITY
香港理工大學

To	Lam Chuen (School of Optometry)		
From	Pang Marco Yiu Chung, Chair, PolyU Institutional Review Board		
Email	marco.pang@polyu.edu.hk	Date	23-Aug-2023

Application for Ethical Review for Teaching/Research Involving Human Subjects

I write to inform you that approval has been given to your application for human subjects ethics review of the following project for a period from 02-Oct-2023 to 02-Oct-2025 **ON CONDITION that appropriate insurance is arranged for the project before the study/trial starts:**

**Project Title:**	Efficacy of a new nanoemulsion artificial tear in dry eye disease management
**Department:**	School of Optometry
**Principal Investigator:**	Lam Chuen
**Project Start Date:**	02-Oct-2023
**Project type:**	Human subjects (clinical)
**Review type:**	Expedited Review
**Reference Number:**	HSEARS20230209004

You are not allowed to start the above project if no appropriate insurance is arranged.

You will be held responsible for the ethical approval granted for the project and the ethical conduct of the personnel involved in the project. In case the Co-PI, if any, has also obtained ethical approval for the project, the Co-PI will also assume the responsibility in respect of the ethical approval (in relation to the areas of expertise of respective Co-PI in accordance with the stipulations given by the approving authority).

You are responsible for informing the PolyU Institutional Review Board in advance of any changes in the proposal or procedures which may affect the validity of this ethical approval.

Pang Marco Yiu Chung

Chair

PolyU Institutional Review Board
