# Supplementary material for: Efficacy of a new nanoemulsion artificial tear in dry eye disease management: Study protocol for a prospective cohort study
Source: PLoS One. 2025 May 9;20(5):e0323523. doi: 10.1371/journal.pone.0323523 (PMC12063801; doi:10.1371/journal.pone.0323523)
Supplement: S4 File — (DOCX) [file pone.0323523.s004.docx]

**Efficacy of** **a new nanoemulsion artificial tear in dry eye disease management**

Ethics committee approved study protocol

**Methodology**

Participant recruitment will be conducted through open recruitment at the university social media platform (Facebook), newspaper advertisement or any other open recruitment method.

All measurements will be conducted on both eyes, and the eye with most severe DED will be chosen and analyzed. The examination flow and instrument used in each assessment are listed in Table 1.

All clinical tests will be administered in accordance with the recommendations of the TFOS DEWS II Diagnostic methodology report[1]. The temperature and humidity of the examination room will be controlled at 23-25^o^C and approximately 50%, respectively. All measurements will be conducted only after having the participants stayed at the examination room for at least 10 minutes.

OSDI questionnaires

OSDI questionnaire will be performed using Qualtrics XM platform.

OSDI questionnaire contains 12 questions with 3 subscales which are ocular symptoms, vision-related function and environmental triggers. Participants are required to rate each symptom on a 5-point scale according to the frequency. The final score is calculated which ranges from 0 to 100. Score between 13-32 will be classified as mild to moderate DED.

Blinking dynamic & quality

SBM idra will be used to measure blinking dynamic and quality. To avoid voluntary blinking, participants will be instructed to look into the instrument and blink normally without alerting them. The blinking dynamic will be video-recorded for 15 seconds with infra-red lighting. Three measurements will be taken and the number and percentage of partial blink will be assessed.

Lipid layer thickness (LLT)

The LLT will be video-documented for 15 seconds using SBM idra. The averaged LLT will be provided. Three video captures will be taken and the LLT measurements will be averaged.

Meniscometry

Three instruments will be used to perform the tear meniscus height (TMH) measurement using SBM idra and Oculus Keratograph 5M. Lower TMH will be measured by averaging three measurements within 1mm of the pupil centre at lower meniscus. Participants will be instructed to look straight during the measurement and the measurement will be captured using Infra-red LED lighting. The caliper tool of the software of each instrument will be used to measure the TMH for each image captured.

Non-invasive tear break-up time (NITBUT / NIKBUT)

Tear film stability and regularity will be assessed using placido disk projection of SBM idra and Oculus Keratograph 5M. Automated detection of the first and average breakup will be generated by the software and readings from three independent captures will be averaged. All measurements will be captured using Infra-red LED lighting.

Tear film osmolarity

Clinical osmometer (Tearlab) will be used to measure the tear film osmolarity. Osmolarity measurement procedure will be in accordance with the manufacturer guideline. In brief, calibration will be performed everyday before use. A tear sample of 50nL will be collected by positioning the tip of the osmolarity pen at the lower lateral canthus tear meniscus. To avoid stimulating the secretion of reflex tear, participants will be instructed to look to the superior nasal direction during measurement. Readings from each eye and the inter-ocular difference will be recorded and accessed.

Tear fluid collection and analysis

Schirmer strip will be used to collect the tear sample. Participants will be asked to look to the superior nasal direction and the strip will be positioned at the lateral canthus. After the strip is placed, participants will be instructed to keep the eyes closed during tear sample collection. A total of 10 – 20ul of tears will be collected. The sampled Schirmer strip will be heated using a optical frame heater to dry out the strip to prevent protein degradation. The dryed stripe will be stored within the 1.5mL Eppendorf tube. Protein assay will be carried out subsequently at the laboratory.

Biomicroscopy

Lid margin, lashes, corneal and conjunctival integrity will be assessed using slit lamp biomicroscopy. Sodium fluorescein and lissamine green dyes will be applied to evaluate corneal and conjunctival desiccations respectively. Staining will be recorded and graded using Oxford scale[2] and lid wiper epitheliopathy will be evaluated using Korb’s grading[3]. Lid and lashes abnormality will also be graded based on a four-point scale. All anterior assessment will be photo-documented.

Infrared meibography

The meibomian glands will be imaged using infrared meibography. Both upper and lower tarsal conjunctiva will be assessed and the evaluation of the meibomian gland will be graded using Meiboscore[4].

*Table 1. Order of assessment and instrument to be used*

| **Order of assessments** | | **Equipment & tools (TBC)** |
| --- | --- | --- |
| 1 | Dry eye questionnaire | OSDI |
| 2 | Blinking dynamic & quality  Tear film lipid layer thickness | Idra (SBM Sistemi) |
| 3 | Meniscometry | Keratograph 5M (Oculus)  Idra (SBM Sistemi) |
| 4 | NITBUT (NIKBUT) | Keratograph 5M (Oculus)  Idra (SBM) |
| 5 | Tear osmolarity | Tearlab (Tearlab) |
| 5 minutes interval | | |
| 6 | Tear fluid collection | Schirmer strip |
| 7 | Biomicroscopy | Slit lamp biomicroscope  NaFl & lissamine green |
| 8 | Infrared Meibography | Keratograph 5M (Oculus)  Idra (SBM Sistemi) |

**Statistical analysis**

Statistic analysis will be conducted using IBM SPSS (New York, USA). Inter-group comparisons of normally distributed continuous measures will be conducted using one way ANOVA tests, followed by Bonferroni corrections. Non-normally distributed continuous measures will be compared using Kruskal-Wallis tests. Ordinal data and categorical data will be compared using Mann-Whitney tests and Fisher’s exact tests respectively. P<0.05 will be considered significant. Univariate and multivariate logistic regression will be conducted to evaluate the relationship between DED and lifestyle factors.

**References**

1. Wolffsohn JS, Arita R, Chalmers R, Djalilian A, Dogru M, Dumbleton K, et al. TFOS DEWS II diagnostic methodology report. 2017;15(3):539-74.

2. Whitcher JP, Shiboski CH, Shiboski SC, Heidenreich AM, Kitagawa K, Zhang S, et al. A simplified quantitative method for assessing keratoconjunctivitis sicca from the Sjögren's Syndrome International Registry. 2010;149(3):405-15.

3. Korb DR, Herman JP, Greiner JV, Scaffidi RC, Finnemore VM, Exford JM, et al. Lid wiper epitheliopathy and dry eye symptoms. 2005;31(1):2-8.

4. Bron A, Benjamin L, Snibson GJE. Meibomian gland disease. Classification and grading of lid changes. 1991;5(4):395-411.
